# Genome-wide association study of idiopathic pulmonary fibrosis susceptibility using clinically curated European ancestry datasets

**DOI:** 10.1183/13993003.00506-2026

**Published:** 2026-07-02

**Authors:** Daniel Chin, Tamara Hernandez-Beeftink, Lauren Donoghue, Beatriz Guillen-Guio, Olivia C. Leavy, Ayodeji Adegunsoye, Helen L. Booth, CleanUP-IPF Investigators of the Pulmonary Trials Cooperative, William A. Fahy, Tasha E. Fingerlin, Bibek Gooptu, Ian P. Hall, Simon P. Hart, Mike R. Hill, Nik Hirani, Simon R. Johnson, Naftali Kaminski, Jose Miguel Lorenzo-Salazar, Shwu-Fan Ma, Robin J. McAnulty, Mark I. McCarthy, Amy D. Stockwell, Toby M. Maher, Ann B. Millar, Philip L. Molyneaux, Maria Molina-Molina, Vidya Navaratnam, Margaret Neighbors, Justin M. Oldham, Helen Parfrey, Gauri Saini, Ian Sayers, X. Rebecca Sheng, Iain D. Stewart, Mary E. Strek, Martin D. Tobin, Moira K.B. Whyte, Maria C. Zarcone, Yingze Zhang, Fernando Martinez, Brian L. Yaspan, Carl J. Reynolds, David A. Schwartz, Carlos Flores, Imre Noth, R. Gisli Jenkins, Richard J. Allen, Louise V. Wain

**Affiliations:** 1 Division of Public Health and Epidemiology, School of Medical Sciences, University of Leicester, Leicester, UK; 2 University Hospitals of Leicester NHS Trust, Leicester, UK; 3 Centre for Fibrosis Research, University of Leicester, Leicester, UK; 4 Genentech, South San Francisco, CA, USA; 5 Centro de Investigación Biomédica en Red de Enfermedades Respiratorias (CIBERES), Instituto de Salud Carlos III, Madrid, Spain; 6 University of Chicago, Chicago, IL, USA; 7 University College London Hospitals, London, UK; 8 Weill Cornell Medicine, New York, NY, USA; 9 GlaxoSmithKline, London, UK; 10 National Jewish Health, Denver, CO, USA; 11 Centre for Respiratory Research, NIHR Nottingham Biomedical Research Centre, School of Medicine, Biodiscovery Institute, University of Nottingham, Nottingham, UK; 12 University of Hull, Hull, UK; 13 University of Oxford, Oxford, UK; 14 University of Edinburgh, Edinburgh, UK; 15 Centre for Respiratory Research, NIHR Biomedical Research Centre and Biodiscovery Institute, Translational Medical Sciences, School of Medicine, University of Nottingham, Nottingham, UK; 16 Yale School of Medicine, New Haven, CT, USA; 17 Genomics Division, Instituto Tecnologico y de Energias Renovables, Santa Cruz de Tenerife, Spain; 18 University of Virginia, Charlottesville, VA, USA; 19 University College London, London, UK; 20 NIHR Imperial Biomedical Research Unit, National Heart and Lung Institute, Imperial College London, London, UK; 21 Keck Medicine of USC, University of Southern California, Los Angeles, CA, USA; 22 University of Bristol, Bristol, UK; 23 National Heart and Lung Institute, Imperial College London, London, UK; 24 Servei de Pneumologia, Hospital Universitari de Bellvitge (HUB), Laboratori de Pneumologia Experimental, Institut de Investigació Biomèdica de Bellvitge (IDIBELL), Barcelona, Spain; 25 Facultat de Medicine, Universitat de Barcelona, Barcelona, Spain; 26 Department of Respiratory Medicine, Sir Charles Gardiner Hospital, Perth, Australia; 27 Centre for Respiratory Research, University of Western Australia, Perth, Australia; 28 University of Michigan, Ann Arbor, MI, USA; 29 Royal Papworth Hospital NHS Foundation Trust, Cambridge, UK; 30 University of Nottingham, Nottingham, UK; 31 Imperial College London, London, UK; 32 University of Pittsburgh, Pittsburgh, PA, USA; 33 UMass Chan Medical School, Worcester, MA, USA; 34 Imperial College, London, UK; 35 University of Colorado Medicine, Aurora, CO, USA; 36 Research Unit, Hospital Universitario Nuestra Señora de Candelaria, Instituto de Investigación Sanitaria de Canarias, Santa Cruz de Tenerife, Spain; 37 Facultad de Ciencias de la Salud, Universidad Fernando Pessoa Canarias, Las Palmas de Gran Canaria, Spain; 38 Contributed equally

## Abstract

Idiopathic pulmonary fibrosis (IPF) is a chronic, progressive lung disease thought to result from an aberrant response to lung injury, culminating in an exaggerated healing response with excessive deposition of extracellular matrix in the interstitium [1].

## Shareable abstract

**New association signals suggest a potentially causal role for the gene encoding KL-6, a known biomarker of fibrosis, and implicate genes involved in angiogenesis in IPF development** https://bit.ly/3Ocr01s

*To the Editor:*

Idiopathic pulmonary fibrosis (IPF) is a chronic, progressive lung disease thought to result from an aberrant response to lung injury, culminating in an exaggerated healing response with excessive deposition of extracellular matrix in the interstitium [1].

Genome-wide association studies (GWAS) have highlighted more than 30 independent genetic signals linked to IPF susceptibility, implicating pathways such as telomere dysfunction, cell–cell adhesion, host defence, transforming growth factor-β signalling, and mitotic spindle assembly [2, 3].

Whole genome sequencing (WGS) technologies provide comprehensive genomic coverage, but remain cost-prohibitive for large studies. Instead, reference panels derived from large WGS datasets allow for improved imputation of unmeasured variants, including those with low allele frequencies, providing a cost-effective solution for increased genome-wide coverage.

Although each genetic signal has a small individual effect, targeting the pathways conferred by genetic associations could offer significant therapeutic potential, as drug targets supported by genetic evidence studies have a higher likelihood of success in clinical development [4].

For this study, we re-imputed previously published studies using a more recent imputation panel that enables measurement of three times as many variants as our previous studies, and aggregated new datasets comprising clinically curated IPF cases, and controls, to discover new loci associated with IPF susceptibility.

Seven independent European ancestry case–control studies were analysed: the Colorado (1515 cases, 4683 UK Biobank controls), USA (541 cases, 542 controls), UK (612 cases, 3365 UK Biobank controls), IPF Job Exposures Study (IPF-JES) (416 cases, 2465 UK Biobank controls), Genentech (813 cases, 3949 controls), USA, UK and Spain (UUS) (793 cases, 9999 UK Biobank controls), and Study of Clinical Efficacy of Antimicrobial Therapy Strategy Using Pragmatic Design in Idiopathic Pulmonary Fibrosis + University of California, Davis (CleanUP-UCD) (469 cases, 2455 UK Biobank controls) datasets. Cases were diagnosed according to the American Thoracic Society and European Respiratory Society guidelines [5]. Written informed consent and ethics approval were properly obtained for all studies, following the World Medical Association's Code of Ethics (Declaration of Helsinki) and approved by the appropriate institutional review board or research ethics committee.

Colorado, IPF-JES, UK, USA, UUS and CleanUP-UCD were genotyped using single nucleotide polymorphism arrays. Quality control measures included filtering for low call rates, sex mismatches, heterozygosity, non-European genetic ancestry, relatedness, and ensuring no overlap between studies, and imputation was performed using the TOPMed WGS Imputation (GRCh38) Server (https://imputation.biodatacatalyst.nhlbi.nih.gov/). Variants with a poor imputation quality (r2 <0.5) and minor allele count ≤3 were removed. Genome-wide association analyses were conducted using logistic regression with PLINK v2 adjusting each study for the first 10 principal components to correct for population stratification. Chromosome X was analysed for UUS, Colorado, CleanUP-UCD and UK, with male and female dosages considered on a 0–2 scale. For Genentech, genotypes were obtained through WGS on the Illumina HiSeq X Ten platform with an average read depth of 30×. Related individuals and those with call rates below 10% were excluded from the analysis. Association analysis was performed using logistic regression with PLINK v1.9 including sex, age, and five genetic-ancestry principal components as covariates. Meta-analysis was performed using inverse-variance weighted fixed effect with METAL (https://github.com/statgen/METAL), excluding variants that did not pass quality control in at least two studies. We estimated the genomic inflation factor using LDSC (https://github.com/bulik/ldsc).

Independent association signals were selected after performing conditional analysis with COJO-GCTA v1.90.2 (https://yanglab.westlake.edu.cn/software/gcta/#COJO) using a threshold of p<5.0×10^−8^. Signals were excluded if the significance (p-value) of the association in any individual study was lower than the meta-analysis significance (*i.e.* if combining data across studies served to reduce the association rather than support it). We sought replication of new signals using an independent subset of the Global Biobank Meta-analysis Initiative (GBMI) IPF GWAS [3] comprising 6257 cases defined using electronic healthcare records (EHR), and 947 616 controls, all of European ancestry (with Bonferroni corrected significance threshold for the number of signals tested).

We analysed 5159 IPF cases and 27 459 controls of European ancestry, and 25 290 839 autosomal variants (figure 1a). For X chromosome, there were 3388 IPF cases and 20 502 controls and 261 736 variants. After conditional analyses, we identified 37 autosomal independent significant signals (p<5×10^−8^). 10 were new, three of which were supported by multiple studies, had not been previously reported and were also replicated in the independent GBMI IPF GWAS (p<0.0125) (figure 1b).

**FIGURE 1:**
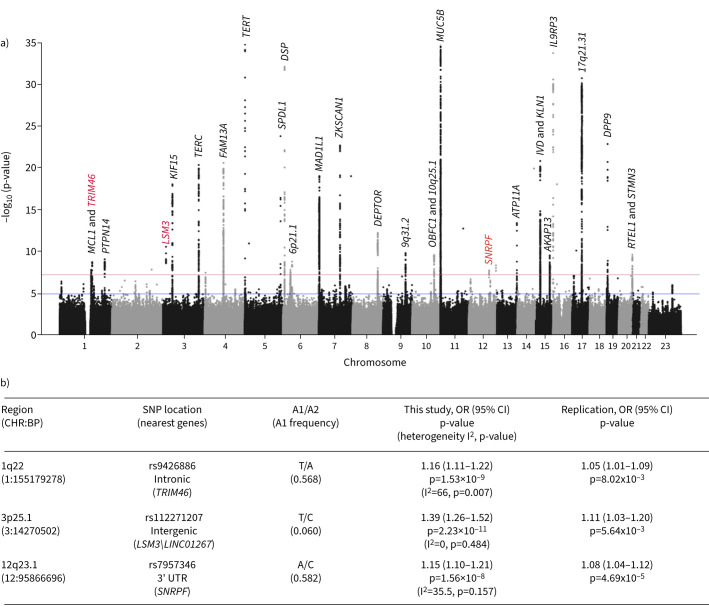
a) Manhattan plot of meta-analysis results. The horizontal red line indicates genome-wide threshold (p=5.0×10^−8^) and blue line indicates suggestive significance threshold (p=5.0×10^−5^). Prior to correction using LDSC intercept, genomic inflation factors were lambda=1.18 (autosomes) and lambda=1.24 (chromosome X). Newly discovered nearest annotated genes are highlighted in red. The plot has been truncated at p=1.00×10^−35^ (*TERT*-rs2736100 p=7.86×10^−36^; *DSP*-rs2076295 p=4.81×10^−53^; *MUC5B*-rs35705950 p=1.39×10^−514^; *IL9RP3*-rs367849850 p=7.82×10^−47^). b) New genome-wide significant association signals. A1: effect allele; A2: non-effect allele; freq.: frequency; CHR: chromosome; BP: base pair position. Coordinates in GRCh38.

The most statistically significant new signal (rs112271207, 3p25.1) was a common (minor allele frequency 6%) intergenic variant located 91.6 kb downstream of *LSM3* and 77.6 kb upstream of *LINC01267*. Another nearby gene, *SLC6A6*, encoding a taurine and beta-alanine transporter gene, has been implicated in hypotaurinemic retinal degeneration and cardiomyopathy [6] and kidney fibrosis in diabetic knockout mice. Taurine may have a role in lung homeostasis and protection against oxidative stress [7], a known driver of IPF pathogenesis.

The second most significant signal (rs9426886, 1q22) was intronic in *TRIM46*, which is involved in the formation of parallel microtubule bundles. This signal is also near to *MUC1*, which encodes a cell surface glycoprotein well known to be important in several lung diseases and infections [8]. The MUC1 glycoprotein comprises three domains; an extracellular domain, a transmembrane domain, and a cytoplasmic tail. The extracellular domain becomes cleaved during alveolar epithelial damage and is commonly known as Krebs von den Lungen-6 (KL-6); a widely recognised potential prognostic biomarker of lung fibrosis that is elevated in the serum and bronchoalveolar lavage fluid of patients with IPF [9]. Another nearby notable gene, thrombospondin-3 (*THBS3*) has a known role in cell-to-cell and cell-to-matrix interactions and has been implicated in cardiac fibrosis [10] and skin healing [11].

Finally, the signal at 12q23.1 (rs7957346) overlaps the 3′-UTR of *SNRPF* and is nearby to *NTN4,* encoding the secreted protein netrin-4. Originally identified as a guide of axon migration, but more recently established to have an important role in kidney and vascular development, and also implicated in lung morphogenesis [12]. The role of netrin-4 in angiogenesis and the renewed interest in the relevance of endothelial cell and vascular abnormalities in IPF [13] make this an intriguing signal for further investigation.

All of the studies included in the genome-wide discovery stage of this analysis comprised cases that have been defined according to clinical criteria. Previous studies have noted an attenuation of effect sizes for IPF risk-associated variants in studies where IPF case status has been defined using routine EHR [3, 14]. Despite a smaller discovery sample size than previous studies that included IPF cases defined by EHR, we identified new signals that were replicated in an independent biobank EHR-based dataset [3].

A key challenge in translating genetic association signals to mechanistic insight is robust variant-to-gene mapping to understand the functional, and ultimately clinical, consequence of the genetic perturbation. Additional functional *in silico* approaches, as well as *in vitro* and *in vivo* studies, are needed to robustly map these new signals to genes, predict their function, and investigate their biological function and relevance to IPF.

Our study only included individuals of European ancestry, which means that we cannot assess the generalisability of these association signals to other populations. Future efforts should be focused on increasing availability of carefully phenotyped IPF datasets from non-European ancestry populations.

In conclusion, we have identified new signals of association with IPF risk that may lead to further mechanistic insight into the pathobiology of IPF and support the identification of potential new drug targets.

## Data Availability

Summary statistics (*i.e.* effect size estimates, standard errors, p-values and basic variant information) for all variants included in the genome-wide meta-analysis can be accessed *via* the GWAS catalogue (www.ebi.ac.uk/gwas/).
